# Legumain level in patients with gestational diabetes to promote ferroptosis through liver gluconeogenesis by HSP90 and GPX4

**DOI:** 10.29219/fnr.v70.12939

**Published:** 2025-12-19

**Authors:** Yingda Yan, Huafang Liu, Xiaodi Kang, Hongli Jiang, Wenjing Wang, Yanli Xu, Fanwen Yang

**Affiliations:** 1 The Third Affiliated Hospital, Guangzhou Medical University, Translational Research Center for Regenerative Medicine and 3D Printing Technologies, Guangzhou Medical University, School of Biomedical Engineering, Guangzhou Medical University, Guangzhou, Guangdong, 510150,China; 2 Department of Obtestric and Gynecology, Beijing Ditan Hospital Affiliated Capital Medical University, Beijing City, 110015, China

**Keywords:** Legumain, gestational diabetes, ferroptosis, GPX4, ubiquitination

## Abstract

**Background:**

The incidence of gestational diabetes mellitus (GDM) is the first diabetes in pregnancy and has gradually increased worldwide, increasing the burden of social healthcare systems. In GDM, oxidative stress induced by reactive oxygen species (ROS) can disrupt the integrity of the cell membrane through lipid peroxidation reactions and trigger ferroptosis, further exacerbating pancreatic islet β-cell dysfunction and insulin resistance. Lipid peroxidation products related to ferroptosis (such as malondialdehyde (MDA) and 4-hydroxynonenal (4-HNE)), along with the decrease in glutathione peroxidase 4 (GPX4) activity, may be involved in the placental oxidative damage and adverse fetal outcomes in GDM, suggesting that targeting the antioxidant pathway or regulating ferroptosis may serve as an intervention strategy. Legumain (LGMN) can serve as novel targets in diabetes mellitus genetic therapy.

**Objective:**

This study investigated the mechanism and effects of LGMN in GDM.

**Method:**

All blood samples of normal or patients with GDM were collected by Beijing Ditan Hospital Affiliated Capital Medical University. C57BL/6J female mice were intraperitoneally injected with streptozotocin. Sh-LGMN virus (20 μg of each) or control vector virus (20 μg of each) was injected into GDM mice. GDM mice randomly assigned to three groups (Number = 10). Sh-LGMN virus (20 μg of each) + HSP90 inhibitor or Sh-LGMN virus (20 μg of each) or control vector virus (20 μg of each) was injected into GDM mice. LGMN or si-LGMN Plasmids were transfected into HepG2 cells using Lipofectamine 2000. HepG2 cells were incubated by different insulin concentrations (100 nmol/L) treatment for 24 h. Microarray analysis, quantitative polymerase chain reaction, enzyme-linked immunosorbent assay, proliferation assay, ethynyl deoxyuridine staining, bioluminescence imaging, and Western blot were used in this study.

**Results:**

Serum LGMN mRNA expression was significantly elevated in patients with GDM. LGMN mRNA and protein expression were also elicited in the liver tissue of GDM mice. LGMN mRNA expression exhibited a positive correlation with Body Mass Index (BMI), fasting plasma glucose, 1-h plasma glucose, or 2-h plasma glucose in patients. Sh-LGMN virus reduced blood glucose levels and body weight, inhibited fasting insulin (FINS) levels and Homeostatic Model Assessment of Beta-cell function (HOMA-β), enhanced Fasting Blood Glucose (FBG)/FINS/Total Cholesterol/Triglycerides levels, improved hepatic fibrosis (HE staining), and also upgraded HbAIc and HOMA-IR in mice of GDM. LGMN exacerbated ROS-induced oxidative stress in the in vitro model of GDM. LGMN promoted ferroptosis in vitro model of GDM. LGMN expanded ROS-induced mitochondrial damage in vitro model of GDM. LGMN inhibited the HSP90/GPX4 Signaling Pathway in the model of GDM. The inhibition of HSP90 reduced LGMN on GDM in the mice model or the in vitro model of GDM. LGMN interlinked with complex protein body of HSP90 and GPX4, which LGMN inhibited the HSP90/GPX4 Signaling Pathway.

**Conclusions:**

The LGMN level in patients with GDM was upregulated, and LGMN facilitates ferroptosis by HSP90 and GPX4 in the mice model of GDM and may lead to therapeutic potential of ferroptosis or liver gluconeogenesis in the model of GDM.

## Popular scientific summary

- LGMN facilitates ferroptosis byHSP90 and GPX4 in the mice model of GDM and may lead to therapeutic potential of ferroptosis or livergluconeogenesis in the model of GDM.

Gestational diabetes mellitus (GDM) is a common pregnancy-specific metabolic disorder. Without timely intervention, GDM can lead to maternal metabolic dysfunction, significantly compromising both maternal health and fetal development (1). Consequently, early prevention and management of GDM is critically important. GDM represents a significant clinical concern, with pathogenesis primarily linked to **insulin resistance** stemming from aberrations in insulin signaling pathways (2). Key independent risk factors include genetic predisposition, glucose intolerance, obesity, and dyslipidemia (3). Research confirms that prolonged hyperglycemia associated with GDM damages vascular endothelial function, thereby elevating the risk of diverse adverse pregnancy outcomes such as premature delivery and miscarriage (3, 4). Therefore, identifying reliable biomarkers for the early screening and diagnosis of GDM is essential for preventing its onset and improving pregnancy outcomes.

Ferroptosis is an iron-dependent form of programmed cell death, distinct from apoptosis, pyroptosis, necrosis, and autophagy (5). This oxidative cell death process results from an imbalance in intracellular lipid reactive oxygen metabolism, diminished cellular antioxidant capacity, and the accumulation of membrane lipid peroxidation products (6). Notably, the ischemia-reperfusion and hypoxia-reoxygenation processes occurring during maternal-fetal interface formation can trigger substantial production of reactive oxygen species (ROS) (7).

Ferroptosis in trophoblasts can impair their invasion and proliferation capabilities. This disruption contributes to inadequate uterine spiral artery remodeling and insufficient placental trophoblast infiltration, ultimately leading to placental dysfunction. Consequently, ferroptosis is implicated in the pathogenesis of placental-related disorders, including pre-eclampsia, preterm delivery, and GDM (8). Ferroptosis in trophoblasts can impair their invasion and proliferation capabilities. This disruption contributes to inadequate uterine spiral artery remodeling and insufficient placental trophoblast infiltration, ultimately leading to placental dysfunction. Consequently, ferroptosis is implicated in the pathogenesis of placental-related disorders, including pre-eclampsia, preterm delivery, and GDM.

Ferroptosis is a distinct, iron-dependent form of programmed cell death, differing fundamentally from apoptosis, necrosis, autophagy, and pyroptosis (9). Its essence is the depletion of GSH and the decrease in glutathione peroxidase 4 (GPX4) activity, resulting in the inability of lipid oxidants to undergo metabolism through the GPX4-catalyzed glutathione reductase reaction (10). This impairment prevents the GPX4-mediated reduction of lipid hydroperoxides, leading to the iron-dependent oxidation of lipids and the accumulation of ROS, which drives the ferroptotic process (11).

Heat shock protein 90 (HSP90) is among the most active chaperones implicated in cancer progression, critically regulating angiogenesis, cell proliferation, migration, invasion, and metastasis (12). HSP90 is overexpressed in numerous malignancies and strongly associated with tumorigenesis and progression – notably, including GDM (13).

LGMN (Legumain), a cysteine endopeptidase, participates in protein processing and exerts diverse biological functions in physiological contexts (14). LGMN is highly expressed in various solid tumors and is closely related to the invasion, diffusion, and metastasis of malignant tumors (15). Its pro-tumorigenic mechanisms involve multifaceted pathways, including modulation of tumor-associated macrophages and neovascular endothelial cells within the tumor microenvironment (16, 17). Previous studies have found that LGMN expression is enhanced renal ischemia-reperfusion injury, suggesting that changes in LGMN expression may play an important role in renal ischemia-reperfusion injury. LGMN can serve as novel targets in diabetes mellitus genetic therapy (18). Therefore, LGMN may be involved in diabetes and its related complications. This study mainly investigated the mechanism and effects of LGMN in the model of GDM.

## Materials & methods

### Patients with GD

This study was approved by the Ethics Committee of Beijing Ditan Hospital Affiliated Capital Medical University (No: 2024126). All the serum samples were immediately snap frozen in liquid nitrogen and stored at –80°C for further using. An informed consent was obtained from all participants. GDM was diagnosed in fasting.

Patients were excluded if they were diagnosed with cardiovascular diseases, cancers or any other major illness, and pregnancies with GDM. Patients who did not fulfill any of the exclusion criteria were recruited in the current study.

GDM was diagnosed in fasting. Basic demographic details of patients are shown in Table 1.

**Table 1:** Basic demographic details of patients

Group	Normal	GDM
Number	26	26
Sex	Female	Female
Age (years)	29.73 ± 3.99	30.04 ± 4.01

GDM: Gestational diabetes mellitus.

### Inclusion and exclusion criteria for clinical studies on GDM

#### 1 Inclusion Criteria

Typically, participants must meet the following criteria:

Confirmed diagnosis of GDM based on internationally recognized diagnostic standards: IADPSG (2013), WHO, Carpenter-Coustan, or NICE guidelines; 100g OGTT (Carpenter-Coustan criteria): Fasting any of the L (95 mg/dL), 1-h ≥ 10.0 mmol/L (180 mg/dL), 2-h ≥ 8.6 mmol/L (155 mg/dL), 3-h ≥ 7.8 mmol/L (140 mg/dL) (diagnosis confirmed if ≥ 2 of the above are abnormal); Gestational age range: Usually 24 confirmed if any of the L (95 mg/dL) rmal)onally recoks; Singleton pregnancy; No pre-pregnancy diabetes; Age ≥ 18 years; Willingness to comply with the study protocol.

75g Oral Glucose Tolerance Test (OGTT, IADPSG criteria), Fasting Blood Glucose Tolerance Test (OGTT, IADPSG, 1-h blood glucose ≥ 10.0 mmol/L (180 mg/dL)), 2-h blood glucose ≥ 8.5 mmol/L (153 mg/dL) (diagnosis confirmed if any of the above is abnormal); 100g OGTT (Carpenter-Coustan criteria): Fasting any of the L (95 mg/dL), 1-h ≥ 10.0 mmol/L (180 mg/dL), 2-h ≥ 8.6 mmol/L (155 mg/dL), 3-h ≥ 7.8 mmol/L (140 mg/dL) (diagnosis confirmed if ≥ 2 of the above are abnormal); Gestational age range: Usually 24 confirmed if any of the L (95 mg/dL) rmal)onally recoks; Singleton pregnancy; No pre-pregnancy diabetes; Age ≥ 18 years; Willingness to comply with the study protocol.

#### 2 Exclusion Criteria

The following participants are typically excluded: Pre-pregnancy diabetes (type 1 or type 2 diabetes); Severe chronic diseases (e.g. uncontrolled hypertension, hepatic or renal insufficiency, cardiovascular diseases, and autoimmune diseases); Multiple pregnancies (twins or more); Gestational age exceeding 32 weeks; Known allergy or intolerance to study drugs (e.g. insulin and metformin); Mental illness or cognitive impairment that affects compliance or the ability to provide informed consent; Participation in other clinical trials that may interfere with this study; Other conditions deemed unsuitable for participation by researchers (e.g. severe infections, malignant tumors, etc.).

### Vivo model of GDM

C57BL/6J female mice (23 ± 2 g; aging 8 ± 2 weeks) were intraperitoneally injected with pentobarbital sodium (40 mg/kg) and streptozotocin (STZ, 40 mg/kg). Sham group of mice were intraperitoneally injected with pentobarbital sodium (40 mg/kg) and normal saline. On the 7th day after injection, Fasting Blood Glucose (FBG) ≥ 11.1 mmol/L was regarded as the successful establishment of the GD mice model. Next, the female mice were pregnant, and GD mice randomly assigned to two groups with 10 mice for each group. Sh-LGMN virus (20 μg of each) or control vector virus (20 μg of each) was injected into GD mice (Figure S1). Finally, GD mice randomly assigned to three groups with 10 mice for each group. Sh-LGMN virus (20 μg of each) + HSP90 inhibitor or Sh-LGMN virus (20 μg of each) or control vector virus (20 μg of each) was injected into GD mice (Fig. S1). All methods were carried out in accordance with relevant guidelines and regulations. All protocols for animals were approved by ethics committee of Beijing Ditan Hospital Affiliated Capital Medical University (NO: 20230723171). All mice were intraperitoneally injected with pentobarbital sodium (40 mg/kg) and sacrificed using the spinal dislocation method.

### Cell culture and transfection

HepG2 cells were performed in compliance with ATCC protocols and incubated in cultured with DMEM/F12 (Gibco, China) routinely supplemented with 10% fetal bovine serum (FBS, PAN3000) at a 5% CO_2_ atmosphere at 37°C. LGMN or si-LGMN Plasmids were transfected into LO2 cell lines using Lipofectamine 2000. HepG2 cells were seeded for 12 h and then incubated in an FBS-free DMEM for 24 h, followed by different insulin concentrations (100 nmol/L) treatment for 24 h as literature (19, 20).

### Microarray analysis

Total RNA was extracted from serum samples, and the amount of RNA was quantified by the use of NanoDrop 1000. Total RNA of each sample was used for reverse transcription using an Invitrogen SuperScript double stranded cDNA synthesis kit. Double-stranded cDNA was executed with a NimbleGen one-color Deoxyribonucleic Acid (DNA) labeling kit and then executed for array hybridization using the NimbleGen hybridization system and washing with the NimbleGen wash buffer kit. The Axon GenePix 4000B microarray scanner (Molecular Devices) was used for scanning.

### Quantitative polymerase chain reaction and enzyme-linked immunosorbent assay

Microarray experiments were performed at the Genminix Informatics (China). Gene expression profiles were analyzed with the Human Exon 1.0 ST GeneChip (Affymetrix). Quantitative polymerase chain reaction (qPCR) was performed with the ABI Prism 7,500 sequence detection system, according to the Prime-ScriptTM RT detection kit. Relative levels of the sample Messenger RNA (mRNA) expression were calculated and expressed as 2^-∆∆Ct^. ROS, MDA (malondialdehyde), SOD, GSH, and GSH-px were detected by using ROS, MDA, Superoxide Dismutase (SOD), Glutathione (GSH), and GSH-px Quantikine ELISA kit. Absorbance at 450 nm was measured using a plate reader as a correction wavelength of 450 nm.

### Proliferation assay and ethynyl deoxyuridine staining

After culturing at indicated time, the cellular proliferation was detected using the MTT assay according to manufacturer’s instructions.

For the ethynyl deoxyuridine (EDU) incorporation assay, EDU (10 mM) was added to each well, and cells were fixed with 4% formaldehyde for 30 min. After washing, EDU was detected using fluorescent microscope (Olympus).

### Bioluminescence imaging

HepG2-hHSP90-Luc were structured according to the previously described (21). Bioluminescent imaging was performed using an IVIS imaging system (Bio-Real, QuickView3000, Austria).

### Western blot

The membranes were incubated with primary antibodies: LGMN (1:1,000, abcam), HSP90 (1:1,000, Cell Signaling Technology, Inc.), GPX4 (1:1,000, abcam), and β-Actin (1:5,000, Santa Cruz Biotechnology) after blocking with 5% BSA in TBS, followed by incubation with peroxidase-conjugated secondary antibodies (Santa Cruz Biotechnology). The signals were detected with the ECL system and exposed by the ChemiDoc XRS system with Image Labsoftware (Bio-rad).

### Statistical analyses

*P* < 0.05 was considered statistically significant using Graphad Prism 6. Comparisons of data between groups were followed using Student’s t test or one-way analysis of variance (ANOVA), followed by Tukey’s post hoc test.

## Results

### The upregulation of LGMN level in patients with GDM

**Fig. 1:**
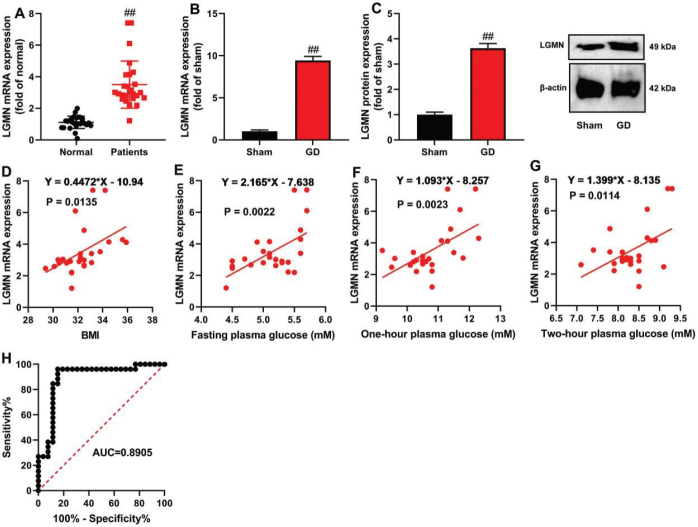
LGMN level in patients with GDM. LGMN mRNA expression (A) was correlation with serum BMI (D), fasting plasma glucose (E), 1 h plasma glucose (F) or 2 h plasma glucose (G), and ROC (h) in patients with GDM; LGMN mRNA (B) and protein (C) expression in mice of GDM. ^##^*P* < 0.01 compared with normal group or Sham group. LGMN: Legumain; GDM: gestational diabetes mellitus; ROC: receiver operating characteristic.

This study first investigated the expression levels of LGMN in GDM. Serum LGMN mRNA expression was significantly elevated in GDM patients (Fig. 1A). Consistently, hepatic LGMN mRNA and protein expression were also upregulated in GDM mouse models (Fig. 1B–C). Clinically, LGMN mRNA expression positively correlated with BMI, fasting plasma glucose, 1 h plasma glucose, or 2 h plasma glucose in patients (Fig. 1D–G). Receiver operating characteristic (ROC) analysis confirmed LGMN’s diagnostic potential for GDM (Fig. 1H).

### The inhibition of LGMN improved glycogen granules in liver cells and GDM in mice of GDM

**Fig. 2:**
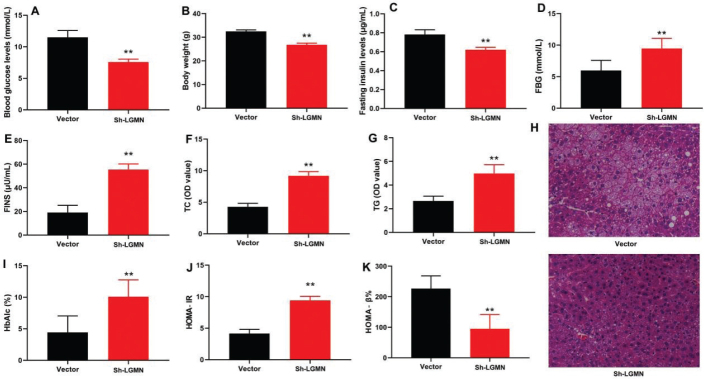
The inhibition of LGMN improved glycogen granules in liver cells and GD in mice of GDM. Blood glucose levels (A), body weight (B), FINS levels (C), FBG activity levels (D)/FINS activity levels (E)/TC activity levels (F)/TG activity levels (G), hepatic fibrosis (HE staining, H), HbAIc (I), HOMA-β (J), and HOMA-IR (K) in mice of GDM. *P* < 0.01 compared with vector group. LGMN: Legumain; FBG: Fasting Blood Glucose; FINS: Fasting Insulin; TC: Total Cholesterol; TG: Triglycerides; HOMA-β: Homeostatic Model Assessment of Beta-cell function.

We determined the effects of LGMN on glycogen granule in mice of GDM. Sh-LGMN virus reduced blood glucose levels and body weight, inhibited fasting insulin (FINS) levels, and Homeostatic Model Assessment of Beta-cell function (HOMA-β), enhanced FBG, FINS, TC (Total Cholesterol), and TG (Triglycerides) levels, improved hepatic fibrosis (HE staining), and also upgraded HbAIc and HOMA-IR in mice of GDM (Fig. 2). Collectively, LGMN inhibition restored hepatic glycogen storage and alleviated GDM progression.

### LGMN expanded ROS-induced oxidative stress in vitro model of GD

**Fig. 3:**
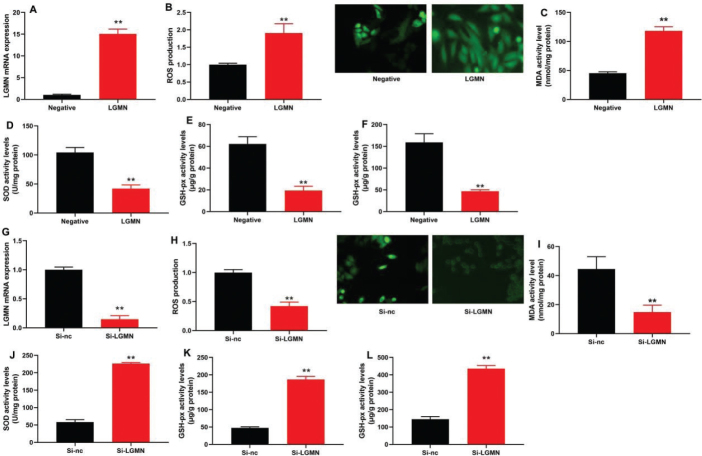
LGMN expanded ROS-induced oxidative stress in vitro model of GDM. LGMN mRNA expression (A), ROS level (B), MDA (C), SOD (D), GSH (E), and GSH-px (F) in vitro model by LGMN upregulation; LGMN mRNA expression (G), ROS level (H), MDA (I), SOD (J), GSH (K), and GSH-px (L) in vitro model by LGMN downregulation. *P* < 0.01 compared with negative or si-nc group. LGMN: Legumain; GDM: gestational diabetes mellitus; ROS: reactive oxygen species; MDA: malondialdehyde.

This study examined the function of LGMN in the glycogen granules in vitro model of GDM. LGMN overexpression (plasmid transfection) increased LGMN mRNA (*P* < 0.001, Fig. 3A) while elevating ROS/MDA (*P* < 0.01, Fig. 3B–C) and reducing antioxidant markers (SOD/GSH/GSH-Px; *P* < 0.05, Fig. 3D–F).

Conversely, LGMN knockdown (siRNA transfection) decreased LGMN mRNA (*P* < 0.001, Fig. 3G) while reducing ROS/MDA (*P* < 0.01, Fig. 3H–I) and enhancing SOD/GSH/GSH-Px (*P* < 0.05, Fig. 3J–L).

### LGMN promoted ferroptosis in vitro model of GDM

**Fig. 4:**
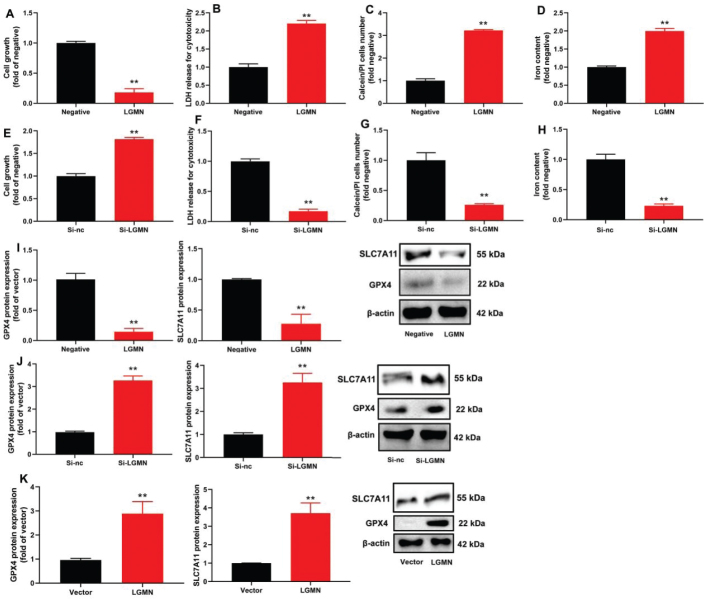
LGMN promoted ferroptosis in vitro model of GDM. Cell proliferation (A), LDH (B), PI positive cells (C), and iron concentration (D) in vitro model by LGMN upregulation; cell proliferation (E), LDH (F), PI positive cells (G), and iron concentration (H) in vitro model by LGMN downregulation; GPX4 protein expression (I and J) in vitro model by LGMN upregulation or LGMN downregulation; GPX4 protein expression (K) in mice of GDM. *P* < 0.01 compared with negative or si-nc or vector group. LGMN: Legumain; GDM: gestational diabetes mellitus; GPX4: glutathione peroxidase 4.

Subsequently, LGMN upregulation reduced cell proliferation and increased Lactate Dehydrogenase (LDH), PI-positive cells, and iron concentration in the in vitro model of GDM (Fig. 4A–D). Conversely, downregulation of LGMN enhanced cell proliferation and decreased LDH, PI-positive cells, and iron concentration in the same in vitro model of GDM (Fig. 4E–H). Meanwhile, in the in vitro model, LGMN upregulation suppressed GPX4/SLC7A11 protein expression, whereas silencing LGMN (si-LGMN) induced their protein expression (Fig. 4I–J). Additionally, in the mouse model, the sh-LGMN virus induced GPX4/SLC7A11 protein expression in the liver tissue of the model mice (Fig. 4K).

### LGMN expanded ROS-induced mitochondrial damage in vitro model of GDM

**Fig. 5:**
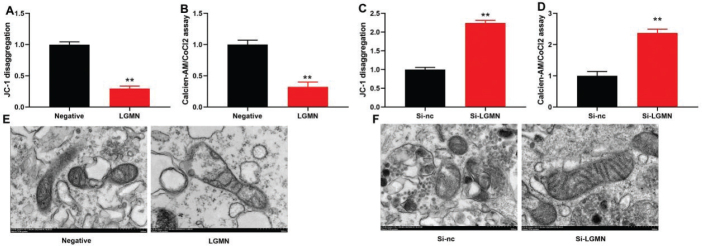
LGMN expanded ROS-induced mitochondrial damage in vitro model of GDM. JC-1 (A), AM-CoCl2 levels (B), and electron microscope (E) in vitro model by LGMN upregulation; JC-1 (C), AM-CoCl2 levels (D), and electron microscope (F) in vitro model by LGMN downregulation. *P* < 0.01 compared with negative or si-nc group. LGMN: Legumain; GDM: gestational diabetes mellitus; ROS: reactive oxygen species.

This study further explored the mechanism by which LGMN regulates ferroptosis in the GDM model. In the in vitro model of GDM, LGMN upregulation reduced the levels of JC-1 and AM-CoCl2, thereby promoting mitochondrial damage (Fig. 5A–B, 5E). In contrast, LGMN downregulation increased the levels of JC-1 and AM-CoCl2 and alleviated mitochondrial damage in the in vitro model of GDM (Fig. 5C–D, F).

### LGMN suppressed HSP90/GPX4 signaling pathway in the model of GD

**Fig. 6:**
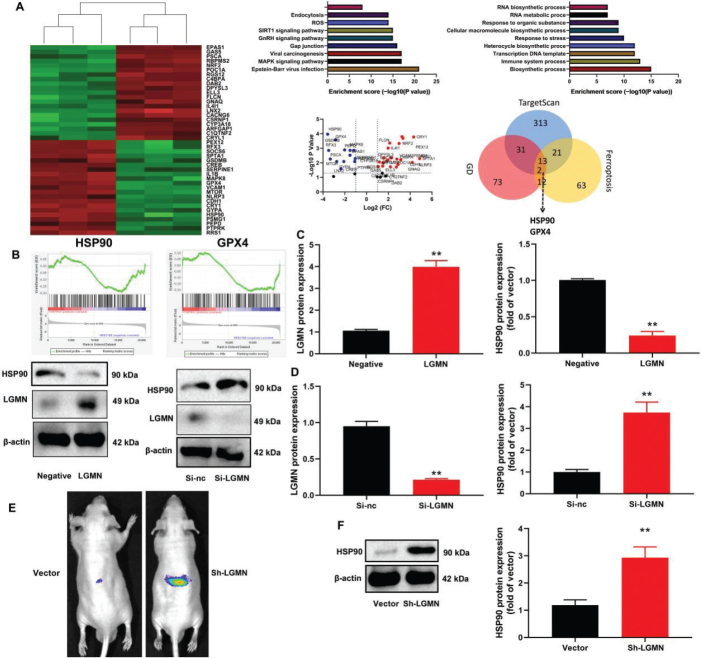
LGMN suppressed HSP90/GPX4 signaling pathway in the model of GDM. Microarray analysis (A), Kyoto Encyclopedia of Genes and Genomes (KEGG) terms (B), and LGMN/HSP90 protein expression (C, D) in vitro model; Bioluminescence imaging (E), HSP90 protein expression in liver tissue (F) in the mice model of GDM. *P* < 0.01 compared with negative or si-nc or vector group. LGMN: Legumain; GDM: gestational diabetes mellitus; HSP90: Heat shock protein 90; GPX4: glutathione peroxidase 4.

We further investigated the potential targets of LGMN in regulating ferroptosis using an in vitro model of GDM. Online bioinformatics tools (TargetScan) and Microarray analysis revealed that HSP90/GPX4 may serve as critical targets of LGMN in GDM models (Fig. 6A–B). In the in vitro GDM model, LGMN upregulation reduced HSP90 protein expression while increasing LGMN protein expression (Fig. 6C). Conversely, LGMN downregulation increased HSP90 protein expression and reduced LGMN protein expression in the same in vitro model (Fig. 6D). Bioluminescence imaging demonstrated that sh-LGMN virus enhanced HSP90 expression in the liver of GDM mice (Fig. 6E). Additionally, sh-LGMN virus induced HSP90 protein expression in the liver tissue of GDM mice (Fig. 6F).

### The inhibition of HSP90 reduced LGMN on GDM in the mice model or in vitro model of GDM

**Fig. 7:**
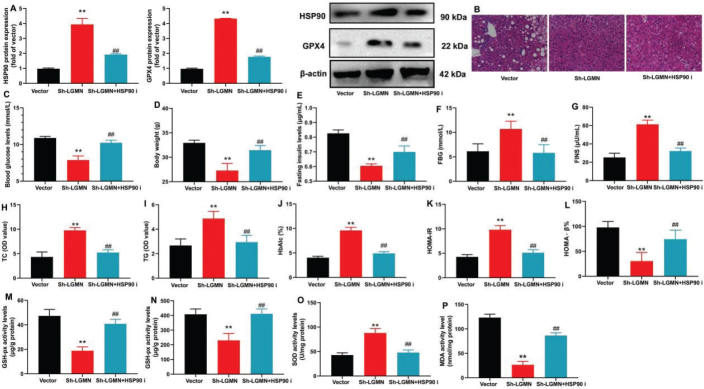
The inhibition of HSP90 reduced LGMN on GDM in the mice model of GD. GPX4/HSP90 protein expression (A), hepatic fibrosis (HE staining, B), blood glucose levels (C), body weight (D), FINS levels (E), FBG activity levels (F)/FINS activity levels (G)/TC activity levels (H)/TG activity levels (I), HbAIc (J), HOMA-β (K), HOMA-IR (L), GSH-px (M), GSH (N), SOD (O), and MDA (P). *P* < 0.01 compared with vector group, ^##^*P* < 0.01 compared with sh-LGMN group. LGMN: Legumain; GDM: gestational diabetes mellitus; HSP90: Heat shock protein 90; GPX4: glutathione peroxidase 4; FBG: Fasting Blood Glucose; FINS: Fasting Insulin; TC: Total Cholesterol; TG: Triglycerides; HOMA-β: Homeostatic Model Assessment of Beta-cell function; MDA: malondialdehyde.

**Fig. 8:**
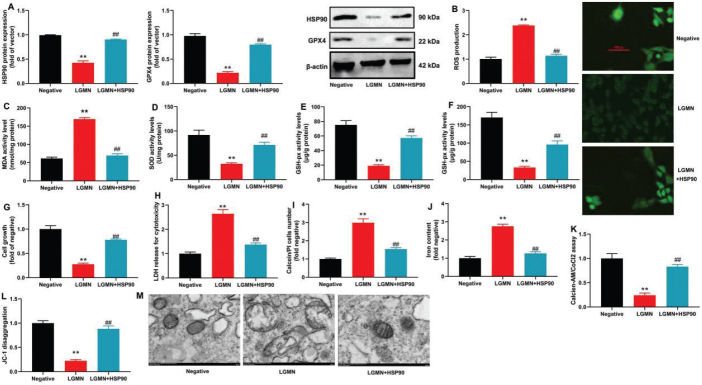
The HSP90 reduced LGMN on the in vitro model of GDM. HSP90/GPX4 protein expressions (A), ROS levels (B), MDA (C), SOD (D), GSH (E), GSH-px (F), cell proliferation (G), LDH (H), PI positive cells (I), iron concentration (J), JC-1 (K), AM-CoCl2 levels (L), and electron microscope (M). *P* < 0.01 compared with negative group, and ^##^*P* < 0.01 compared with LGMN group. LGMN: Legumain; GDM: gestational diabetes mellitus; HSP90: Heat shock protein 90; GPX4: glutathione peroxidase 4; ROS: reactive oxygen species; MDA: malondialdehyde.

**Fig. 9:**
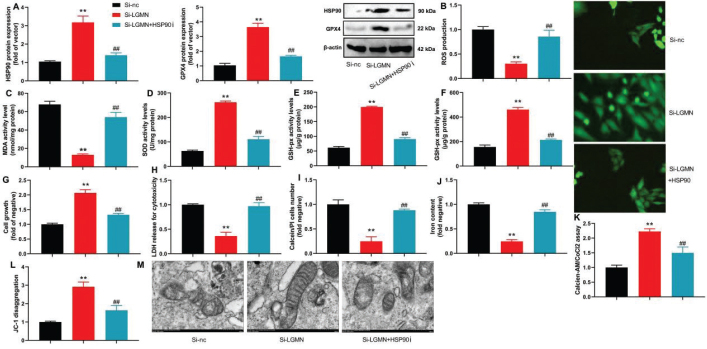
The inhibition of HSP90 reduced si-LGMN on the GDM in vitro model of GDM. HSP90/GPX4 protein expressions (A), ROS levels (B), MDA (C), SOD (D), GSH (E), GSH-px (F), cell proliferation (G), LDH (H), PI positive cells (I), iron concentration (J), JC-1 (K), AM-CoCl2 levels (L), and electron microscope (M). *P* < 0.01 compared with si-nc group, and ^##^*P* < 0.01 compared with si-LGMN group. LGMN: Legumain; HSP90: Heat shock protein 90; GPX4: glutathione peroxidase 4; ROS: reactive oxygen species; MDA: malondialdehyde.

Subsequently, we explored the role of LGMN in regulating HSP90 in both mouse and in vitro GDM models. In the mouse GDM model, treatment with the HSP90 inhibitor, Alvespimycin (100 mg/kg), attenuated the effects of sh-LGMN virus on HSP90/GPX4 protein expressions, glycogen granules, and oxidative stress (Fig. 7). Meanwhile, in the in vitro GDM model, the HSP90 agonists (Tamoxifen, 0.5 μM) also reduced the impact of LGMN on HSP90/GPX4 protein expressions, ROS-induced oxidative stress, ferroptosis, and mitochondrial damage in the in vitro model of GDM (Fig. 8). Similarly, the HSP90 inhibitor Alvespimycin (50 nM) reversed the effect of si-LGMN on HSP90/GPX4 protein expressions, ROS-induced oxidative stress, ferroptosis, and mitochondrial damage in the in vitro GDM model (Fig. 9).

### LGMN interlinked with complex protein body of HSP90 and GPX4

**Fig. 10:**
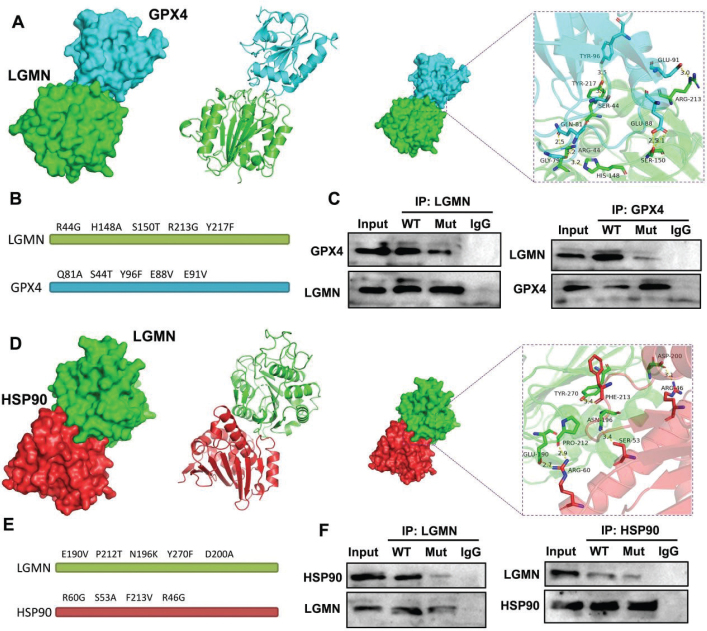
LGMN interlinked with complex protein body of HSP90 and GPX4. The binding energy between GPX4 and LGMN (A), WT/Mut of GPX4 and LGMN (B), LGMN interlinked with complex protein body of GPX4 (C), the binding energy between HSP90 and LGMN (D), WT/Mut of HSP90 and LGMN (E), and LGMN interlinked with complex protein body of HSP90 (F). LGMN: Legumain; HSP90: Heat shock protein 90; GPX4: glutathione peroxidase 4.

Finally, we investigated the mechanism of which LGMN regulates the protein expression of HSP90/GPX4. To explore this, we retrieved the protein structures of GPX4 (PDB ID: 6HN3), HSP90 (PDB ID: 6U98), and LGMN (PDB ID: 8AE4) from the PDB database and performed molecular docking using Hdock. The resulting interaction patterns were analyzed using Pymol 2.3.0 in the interactive mode. The docking results indicate that the binding energy between GPX4 and LGMN is –259.3 kcal/mol. Several residues surrounding the protein-protein interaction interface can form hydrogen bonds (Fig. 10A). Co-immunoprecipitation (IP) assays demonstrated that LGMN interacted with the GPX4 protein complex; however, no interaction was observed between LGMN and the mutant GPX4 (GPX4 Mut) complex nor between GPX4 and the mutant LGMN (LGMN Mut) complex (Fig. 10B–C). Additionally, docking results revealed a binding energy of –257.58 kcal/mol between LGMN and HSP90 (Fig. 10D). IP assays further confirmed that LGMN interlinked with the HSP90 protein complex, while no interaction was detected between LGMN and the mutant HSP90 (HSP90 Mut) complex or between HSP90 and the mutant LGMN (LGMN Mut) complex (Fig. 10F–G).

## Discussion

GDM refers to abnormal glucose metabolism that occurs during pregnancy. Its overall prevalence worldwide is as high as 17.8%, and in China, it ranges from 15 to 20%, with the prevalence increasing year by year (6). GDM not only poses multiple risks to the health of pregnant women themselves, such as gestational hypertension, polyhydramnios, and postpartum hemorrhage but also has a severe impact on fetal growth and development, which can lead to adverse pregnancy outcomes, including macrosomia, fetal distress, and preterm birth (22). Glycolipid metabolism disorder is one of the main characteristics of GDM (23). In-depth research on nutritional support strategies for GDM and their relationship with glycolipid metabolism disorder is of great significance for improving maternal and infant health and reducing the incidence of adverse pregnancy outcomes (24). The main feature of GDM is the glucose metabolism disorder. Elevated hormone levels in pregnant women lead to insulin resistance, thereby causing an increase in blood glucose. Insulin resistance reduces insulin sensitivity, increases fat decomposition, and promotes the release of free fatty acids. Therefore, GDM patients are often accompanied by lipid metabolism disorder. With the growing emphasis on maternal health during pregnancy, the management of GDM has become an important issue in the field of obstetrics (25). In this study, we observed upregulated serum LGMN mRNA levels in patients with GDM. Consistently, elevated LGMN mRNA and protein expression were also detected in the liver tissue of GDM mice. Choi et al. showed that LGMN represents a potential therapeutic target for diabetes mellitus genetic therapy (18). Therefore, our data indicate that LGMN might be participated in the disease progression of GDM. The reason for the sudden increase of LGMN in GDM disease is still unclear. This is also a limitation of this study and an important issue that needs to be addressed in the next experimental step.

As gestational age advances, fetal nutritional demands increase, while maternal blood glucose levels typically decrease (26). Concurrently, elevated levels of sex hormones, cortisol, and other insulin-resistant active factors induce varying degrees of insulin resistance in pregnant women. This triggers compensatory hyperinsulinemia, contributing to the development of GDM (27). Failure to implement scientific dietary control during this period readily induces GDM, which substantially compromises maternal health and pregnancy outcomes (28). Although nutritional intervention – a widely adopted specific management strategy during pregnancy – cannot fully resolve GDM, it offers significant clinical benefits in improving maternal quality of life (29). Here, our data demonstrated that LGMN inhibition improved hepatic glycogen storage and attenuated gestational diabetes in GDM model mice. Chen et al. indicated that LGMN deficiency reduced ROS and MDA levels in acute kidney injury (16). Collectively, these results indicate that LGMN contributes to impaired glycogen metabolism in GDM. This study only aims to inhibit the level of LGMN in a mouse model by Sh-LGMN. This is a limitation of our study. In the next step of research, we will use gene knockout mice or other techniques to regulate the level of LGMN in mouse models to validate our conclusions.

Ferroptosis, a novel programmed cell death pathway with distinct morphological, metabolic, and genetic characteristics, plays significant roles in diverse pathologies, including tumors, neurodegenerative disorders, ischemia-reperfusion injury, atherosclerosis, diabetes, and acute/chronic kidney disease (30). Crucially, placental trophoblasts exhibit heightened susceptibility to ferroptosis, where trophoblastic ferroptosis drives placental dysfunction and contributes to placental-related disease pathogenesis (31, 32).

Thus, our findings establish LGMN-mediated potentiation of mitochondrial damage and ferroptosis as a key mechanism in GDM pathogenesis.

In our GDM models, LGMN exacerbated ROS-induced mitochondrial damage and promoted ferroptosis. Supporting this mechanistic link, Chen et al. reported that LGMN deficiency attenuates ferroptosis in acute kidney injury (16). Thus, our findings establish LGMN-mediated potentiation of mitochondrial damage and ferroptosis as a key mechanism in GDM pathogenesis. This study only found that LGMN promotes ferroptosis induced by mitochondrial oxidation. However, the conclusions of this study have not been verified using relevant inhibitors (e.g. ferrostatin-1 and liproxstatin-1). In the next step of the research, we will explore the role of LGMN using ferroptosis inhibitors and ROS inhibitors.

Ferroptosis – a recently characterized form of programmed cell death – exhibits distinct genetic, biological, and morphological features compared to apoptosis, autophagy, and necrosis. It is mechanistically linked to ischemia-reperfusion injury pathogenesis (33), primarily triggered by Xc-system inhibition and GPX4 (34). Key drivers include excessive ROS release, GPX4 dysfunction, and intracellular lipid peroxide accumulation (35). In our GDM models, LGMN suppressed the HSP90/GPX4 signaling axis. This aligns with Chen et al.’s report that LGMN deficiency elevates GPX4 protein levels in acute kidney injury (16). Collectively, these results indicate that LGMN suppressed HSP90/GPX4 signaling pathway to promote ferroptosis in the model of GDM.

Mechanistically, Hsp90 – a highly conserved molecular chaperone – ensures proper protein folding/stability under stress (e.g. thermal/oxidative) via client protein interactions (36). Its isoforms (HSP90α/β) localize to endothelial cells, cytoskeletons, and nuclei (37, 38). Critically, we identified LGMN as a novel interactor within the HSP90-GPX4 protein complex. Supporting this, Chen et al. observed LGMN deficiency induces HSP90 expression in kidney injury (16). Thus, LGMN-mediated disruption of the HSP90-GPX4 complex represents a key ferroptosis induction mechanism in GDM pathogenesis.

**Fig. 11:**
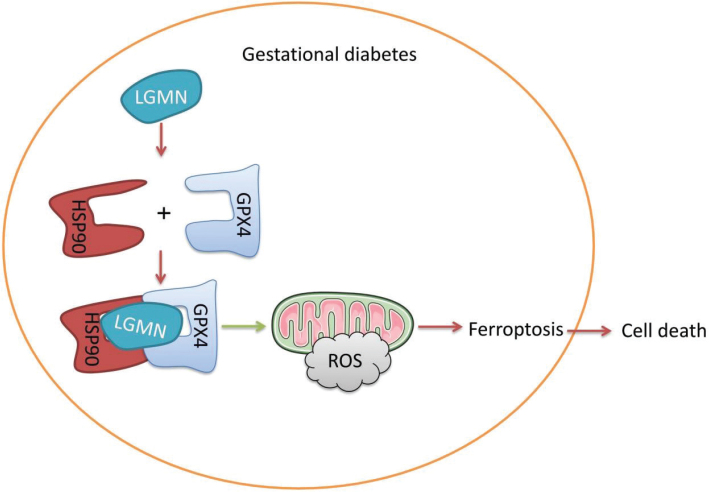
LGMN level in patients with gestational diabetes to promote ferroptosis through liver gluconeogenesis by HSP90 and GPX4. LGMN: Legumain; HSP90: Heat shock protein 90; GPX4: glutathione peroxidase 4.

In conclusion, this article assumes that LGMN may regulate diabetes and its complications. We explore its role and mechanism. Through experiments, LGMN levels in patients with GDM were upregulated. Our results here suggest that LGMN promoted ferroptosis through liver gluconeogenesis by HSP90 and GPX4 signaling pathway (Fig. 11). LGMN may lead to therapeutic potential of ferroptosis or liver gluconeogenesis in the model of GDM. LGMN may become a potential clinical treatment target.

## Declarations

### Ethics approval and consent to participate

The current study was approved by the Ethics Committee of Beijing Ditan Hospital Affiliated Capital Medical University. This study was complied with the guidelines outlined in the declaration of Helsinki. The written consent was received from all participants.

## Data Availability

The datasets used and/or analyzed during the current study are available from the corresponding author upon reasonable request.
